# Stoma versus no stoma prior to long-course neoadjuvant therapy in rectal cancer

**DOI:** 10.1093/bjsopen/zrae169

**Published:** 2025-03-18

**Authors:** Gustav Sandén, Petrus Vinnars, Ingrid Ljuslinder, Johan Svensson, Martin Rutegård

**Affiliations:** Department of Diagnostics and Intervention, Surgery, Umeå University, Umeå, Sweden; Department of Diagnostics and Intervention, Surgery, Umeå University, Umeå, Sweden; Department of Diagnostics and Intervention, Oncology, Umeå University, Umeå, Sweden; Umeå School of Business, Economics and Statistics, Umeå University, Umeå, Sweden; Department of Diagnostics and Intervention, Surgery, Umeå University, Umeå, Sweden

## Abstract

**Background:**

Large bowel obstruction is a possible complication in patients undergoing neoadjuvant treatment for rectal cancer; however, it may be prevented by placing a pretreatment defunctioning stoma. The aim of this retrospective study was to investigate complication rates in patients with rectal cancer undergoing long-course neoadjuvant therapy, comparing those with and without a prophylactic stoma.

**Methods:**

All patients with rectal cancer undergoing neoadjuvant therapy between 2007 and 2022 in Region Västerbotten, Sweden, were identified using the Swedish Colorectal Cancer Registry. Patients not planned for curative long-course neoadjuvant therapy and those requiring a stoma due to urgent bowel-related issues before treatment were excluded. The primary outcome was the incidence of complications between diagnosis and resection surgery or end of follow-up. The secondary outcomes were 30-day complications following resection, time to treatment (neoadjuvant therapy and surgery), and overall survival. Multivariable regression analysis was used, with adjustment for age, sex, American Society of Anesthesiologists fitness grade, and clinical tumour stage.

**Results:**

Of 482 identified patients, 105 were analysed after exclusion. Among these, 22.9% (24 of 105) received a pretreatment stoma, whereas 77.1% (81 of 105) received upfront neoadjuvant therapy. The complication incidence before resection in the group with a defunctioning stoma and in the group without a defunctioning stoma was 75.0% (18 of 24) and 29.6% (24 of 81) respectively. A considerable number of complications were directly caused by the stoma surgery. Patients in the stoma group had an adjusted OR of 6.71 (95% c.i. 2.17 to 20.76) for any complication. However, for 30-day complications following resection, an adjusted non-significant OR of 2.05 (95% c.i. 0.62 to 6.81) was documented for the stoma group, in comparison with the control group. Neoadjuvant treatment was also delayed for the stoma group (adjusted mean time difference: 21 (95% c.i. 14 to 27) days), whereas the difference was not significant for the time to resection surgery. The median survival after diagnosis was 4.7 years in the stoma group and 12.2 years in the control group (*P* = 0.015); however, adjustment in the multivariable analysis rendered the estimate non-significant (HR 1.71 (95% c.i. 0.93 to 3.14)).

**Conclusion:**

Patients with rectal cancer who receive a stoma before long-course neoadjuvant therapy, in the absence of urgent symptoms, experience more complications than those without a stoma and a delay with regard to the start of neoadjuvant treatment.

## Teaser

This population-based cohort study represents the largest report to date on the implementation of prophylactic stomas before neoadjuvant therapy in rectal cancer. While these stomas are commonly used, they may result in increased morbidity before resection, including severe complications directly attributable to the stoma surgery.

## Introduction

Patients with rectal cancer with an intermediate or high risk of locoregional recurrence after surgery are currently treated using neoadjuvant treatment, consisting of radiotherapy and/or chemotherapy^1,2^.

The duration of the neoadjuvant treatment varies, but often spans several months^3^. In Sweden, treatment regimens have evolved over time. So-called total neoadjuvant therapy using the RAPIDO regimen, 5.0 Gy of radiation delivered daily for 5 consecutive days followed by four to six cycles of chemotherapy, results in a higher pCR rate and a decreased risk of distant metastasis compared with the previous standard regimen of 50.4 Gy administered in fractions of 1.8–2.0 Gy alongside concomitant chemotherapy^4,5^. One major disadvantage with either regimen is the prolonged time interval between diagnosis and resection surgery, during which complications may arise that could interfere with the neoadjuvant therapy or the subsequent surgical procedure^6^.

The surgeon may opt to create a defunctioning stoma before initiation of neoadjuvant treatment to prevent certain tumour-related complications, such as large bowel obstruction or perforation^7^. However, such stomas could in turn cause numerous postoperative or stoma-related complications^8^. The available literature on this topic is limited, primarily consisting of small-scale, single-centre studies. Nevertheless, pretreatment defunctioning stomas seem to be in widespread use, even in patients without signs of bowel obstruction^9^.

The aim of this study was to investigate whether the creation of a defunctioning stoma before long-course neoadjuvant therapy is beneficial for patients with rectal cancer who do not have clear indications for such a stoma.

## Methods

### Ethics approval

The Swedish Ethical Review Authority (dnr 2021-01416 with complementary dnr 2023-05853-02) approved this study.

### Patient consent

The need for informed consent from participants was waived by the review board due to the retrospective nature of the study, with no planned intervention; thus, informed consent was not obtained. Moreover, some of the participants have died and any attempts to approach relatives for consent were deemed to incur more harm than good. In addition, all data are presented at a group level, thus making it impossible to identify individual patients.

### Study design

This was a population-based, retrospective cohort study of patients with rectal cancer diagnosed between 2007 and 2022 in Region Västerbotten, Sweden. Patients were identified using the Swedish Colorectal Cancer Registry, from which those undergoing neoadjuvant therapy were extracted^10^. Clinical and demographic data were obtained from the registry, as well as from medical records. Patients were followed up until 15 November 2023.

### Registry data

The Swedish Colorectal Cancer Registry defines rectal cancer as an adenocarcinoma with an inferior margin within 15 cm of the anal verge, measured by rigid sigmoidoscopy. The following data were obtained from the registry: sex (male/female), fitness level (graded using the American Society of Anesthesiologists (ASA) classification), age at diagnosis (years, as a continuous variable), dates of diagnosis/resection surgery/death, tumour height (centimetres from the anal verge), tumour stage (both clinical and pathological, following the tumour node metastasis (TNM) classification system), and surgical resection technique^11,12^. All missing registry data were manually retrieved from patient charts.

### Data extracted and exposures

Medical records were scrutinized in two stages. First, the type and length of the neoadjuvant treatment were noted and patients who received only short-course radiotherapy (25.0 Gy given in fractions of 5.0 Gy for 5 consecutive days) without any chemotherapy were excluded. The remaining patients were treated with neoadjuvant regimens lasting for at least 2 months. Second, these patients were then reviewed in further detail regarding symptoms present at the time of diagnosis, the establishment of any defunctioning stoma before neoadjuvant treatment (including the type of stoma utilized and its rationale, if applicable), and complications (surgical and non-surgical) before resection surgery and within 30 days following resection. Complications were assessed according to the extended Clavien–Dindo classification of surgical complications, but only complications classified as greater than or equal to Clavien–Dindo grade II were considered^13^. Non-surgical complications, such as toxicity that necessitated total parenteral nutrition or opportunistic infections caused by chemotherapy-induced immunosuppression, were evaluated using the same classification system to facilitate accurate comparisons across groups. The highest graded complication for each patient was noted, with particular emphasis on any complication greater than or equal to Clavien–Dindo grade IIIa directly attributable to the stoma surgery, or absence thereof. Patients who progressed to an incurable state during neoadjuvant treatment, resulting in omission of resection surgery, were followed until death regarding complications.

Patients were excluded when they did not match the target demographic regarding region and diagnosis, were classified as incurable shortly after diagnosis, had recurrent disease, had a stoma before diagnosis, or demonstrated clear indications for a defunctioning stoma at the time of diagnosis. Patients within the latter category were evaluated on a case-by-case basis and the rationale for their exclusion was documented separately. Patients with metastatic disease were included when treated with curative intent. Exposed patients were those who received a planned defunctioning stoma before initiation of neoadjuvant treatment (the stoma group), whereas patients who did not receive such a stoma constituted the control group.

### Outcomes of interest

The primary outcome of interest was the incidence of complications occurring between diagnosis and resection surgery. The secondary outcomes were complications within 30 days following cancer resection, time to treatment (neoadjuvant therapy and resection surgery respectively), and overall survival.

### Statistical analysis

The number and proportion of planned defunctioning stomas created before long-course neoadjuvant therapy are described as a function of time. Baseline characteristics between groups were compared using the Mann–Whitney *U* test for continuous variables and Fisher’s exact test for categorical variables. The Mann–Whitney *U* test was also used to make inferences about the complication incidence across groups, and complications of particular interest with regard to the study question are presented in detail for descriptive purposes. Time-to-event data were evaluated using the *t* test and the log rank test. Furthermore, the mean difference in time to neoadjuvant treatment between the groups was analysed using linear regression and visualized using box plots, whereas Kaplan–Meier curves are used to describe time to resection surgery and survival. Multivariable logistic regression (outcome dichotomized to the occurrence or not of complications greater than or equal to Clavien–Dindo grade II) and Cox regression (proportional hazard assumption evaluated visually) models were employed for confounding adjustment, where ORs and HRs respectively, along with 95% confidence intervals, were estimated. The same adjustments were made to the linear regression model used to analyse time to neoadjuvant treatment. The covariates included in these analyses were age (continuous), sex (male or female), ASA fitness grade (I, II, or III–IV), and clinical tumour stage (I–II, III, or IV). For the primary outcome, sensitivity analyses were conducted using resected patients only, as well as dichotomizing the outcome to the occurrence or not of complications greater than or equal to Clavien–Dindo grade IIIa. A complete-case approach was used throughout, as there were no missing data. For all analyses, the statistical software STATA (StataCorp, Collage Station, TX, USA; version 18.0) was used.

## Results

### Patients

**Fig. 1:**
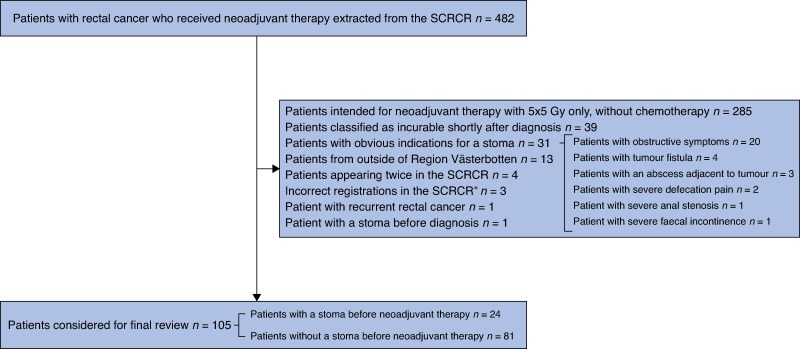
Study flow chart Excluded patients are shown in the middle box. Once a patient was found to fulfil an exclusion criterion, that patient was not reviewed further. Thus, excluded patients may have fulfilled other exclusion criteria as well. *One patient with transverse colon cancer, one patient with sigmoid colon cancer, and one patient with high-grade rectal dysplasia. SCRCR, Swedish Colorectal Cancer Registry.

In total, 482 identified patients were diagnosed with rectal cancer and underwent neoadjuvant therapy between 2007 and 2022 in Region Västerbotten, Sweden. After the application of exclusion criteria, 105 patients remained with a median follow-up time of 4.8 (interquartile range (i.q.r.) 2.4–9.0) years. The study flow chart in *Fig. 1* provides details regarding the exclusion process, including the various conditions mandating a pretreatment stoma in 31 excluded patients. Among the retained patients, 24 received a planned stoma before neoadjuvant therapy, whereas the remaining 81 did not. Most patients in the stoma group received a sigmoid colostomy (16 patients), although ileostomies (4 patients) and transverse colostomies (4 patients) were also used.

Demographic and clinical details of those with and without a pretreatment defunctioning stoma are presented in *Table 1*. The two groups exhibited relatively similar characteristics in terms of sex, ASA fitness grade, and tumour height. However, a higher proportion of stage III cancers was observed in the control group and a higher proportion of stage IV cancers was observed in the stoma group. This pattern persisted in the pathological tumour stage, with the additional observation that the stoma group did not include any pathological complete responders or any stage I cancers. The type of neoadjuvant therapy administered differed considerably, as nearly all of the patients who received short-course radiotherapy followed by chemotherapy were in the control group, whereas most patients in the stoma group received long-course radiotherapy (with or without concomitant chemotherapy). One patient in the stoma group originally intended for long-course radiotherapy ultimately underwent short-course radiotherapy due to suffering a type 2 myocardial infarction following the stoma surgery. Regarding the type of resection surgery performed, abdominoperineal excision was the predominant technique used in the stoma group, whereas anterior resection was most commonly used in the control group. A total of eight patients were classified as incurable due to disease progression following neoadjuvant therapy, leading to the abandonment of the planned resection surgery. Additionally, one patient in the control group had, at the end of follow-up, not undergone resection surgery due to a cCR following neoadjuvant therapy, instead being monitored using a watch-and-wait protocol.

**Table 1:** Baseline characteristics of 105 patients who received long-course neoadjuvant therapy for rectal cancer

	Patients without a stoma before neoadjuvant therapy, *n* = 81	Patients with a stoma before neoadjuvant therapy, *n* = 24	*P*
Age (years), median (i.q.r.)	65 (56–71)	67 (59–75)	0.336
**Sex**			0.358
Male	45 (55.6)	16 (66.7)	
Female	36 (44.4)	8 (33.3)	
**ASA fitness grade**			0.404
I	14 (17.3)	4 (16.7)	
II	53 (65.4)	13 (54.2)	
III–IV	14 (17.3)	7 (29.2)	
**Tumour height (cm)**			0.948
<6	35 (43.2)	11 (45.8)	
7–12	36 (44.4)	11 (45.8)	
13–15	10 (12.3)	2 (8.3)	
**Clinical tumour stage**			0.137
I	0 (0.0)	0 (0.0)	
II	12 (14.8)	6 (25.0)	
III	52 (64.2)	10 (41.7)	
IV	17 (21.0)	8 (33.3)	
**Planned stoma type**			
Ileostomy		4 (16.7)	
Transverse colostomy		4 (16.7)	
Sigmoid colostomy		16 (66.7)	
**Type of long-course neoadjuvant treatment**			<0.001
Long-course radiotherapy with concomitant chemotherapy	36 (44.4)	13 (54.2)	
Short-course radiotherapy followed by chemotherapy	31 (38.3)	1 (4.2)	
Chemotherapy followed by short-course radiotherapy	9 (11.1)	1 (4.2)	
Long-course radiotherapy only	2 (2.5)	7 (29.2)*	
Chemotherapy only	3 (3.7)	2 (8.3)	
**Resection surgery type**			0.017
Anterior resection	38 (46.9)	5 (20.8)	
Abdominoperineal excision	36 (44.4)†	12 (50.0)	
Hartmann’s procedure	2 (2.5)	3 (12.5)	
No resection surgery	5 (6.2)	4 (16.7)	
**Pathological tumour stage**			0.194
pCR	5 (6.2)	0 (0.0)	
I	4 (4.9)	0 (0.0)	
II	15 (18.5)	7 (29.2)	
III	34 (42.0)	6 (25.0)	
IV	18 (22.2)	7 (29.2)	
No resection surgery	5 (6.2)	4 (16.7)	

Values are *n* (%) unless otherwise indicated. Statistical comparisons were performed using the Mann–Whitney *U* test for continuous variables and Fisher’s exact test for categorical variables. *One of these patients was intended for long-course radiotherapy, but ultimately received short-course radiotherapy due to complications following the stoma surgery. †One of these patients had a total proctocolectomy. i.q.r., interquartile range; ASA, American Society of Anesthesiologists.

### Complications

As shown in *Table 2*, there was a significantly higher incidence and severity of complications overall in the stoma group compared with the control group during the interval between diagnosis and resection surgery (Mann–Whitney *U* test: *P* < 0.001). The adjusted OR for any complication in the stoma group, compared with the control group, was 6.71 (95% c.i. 2.17 to 20.76) and similar findings were demonstrated when excluding patients without subsequent resection surgery (*Table 3*). The elevated complication rate in the stoma group was mostly attributed to complications greater than or equal to Clavien–Dindo grade IIIa; in a sensitivity analysis, the adjusted OR for complications greater than or equal to Clavien–Dindo grade IIIa was 12.26 (95% c.i. 3.14 to 47.92). Moreover, five of these complications were directly caused by the stoma surgery: reoperation due to bleeding; drainage of a postoperative abscess; skin grafting following postoperative wound infection; postoperative septic shock; and postoperative myocardial infarction. However, it is worth noting that three patients in the control group suffered large bowel obstruction. One of these patients presented during neoadjuvant therapy, necessitating an emergency stoma that prevented the remainder of the planned chemotherapy from being administered. The second patient underwent endoscopic dilation a few days before liver metastasis surgery, during which a stoma was also created to prevent further obstruction. The third patient suffered bowel perforation, albeit several months after being deemed incurable due to metastatic disease progression.

**Table 2:** Complications between diagnosis and resection surgery in 105 patients with rectal cancer who received long-course neoadjuvant therapy

Clavien–Dindo grade	Patients without a stoma before neoadjuvant therapy, *n* = 81		Patients with a stoma before neoadjuvant therapy, *n* = 24	
	Complications overall, *n* (%)	Complications likely to be avoided had a stoma been created, *n* (% of overall)	Complications overall, *n* (%)	Complications directly related to the stoma, *n* (% of overall)
<II	57 (70.4)		6 (25.0)	
II	18 (22.2)	0 (0.0)	7 (29.2)	3 (42.9)
IIIa	0 (0.0)	NA	3 (12.5)	1 (33.3)
IIIb	1 (1.2)	1 (100.0)	2 (8.3)	2 (100.0)
IVa	1 (1.2)	0 (0.0)	1 (4.2)*	1 (100.0)*
IVb	0 (0.0)	NA	1 (4.2)	1 (100.0)
V	4 (4.9)	0 (0.0)	4 (16.7)	0 (0.0)

Only the highest graded complication for each patient was noted. Mann–Whitney *U* test: *P* < 0.001 (complications overall). *This patient was intended for long-course radiotherapy, but ultimately received short-course radiotherapy due to complications following the stoma surgery. NA, not applicable.

**Table 3:** Multivariable logistic regression analysis: unadjusted and adjusted ORs with 95% confidence intervals for 105 patients with rectal cancer who received long-course* neoadjuvant therapy, using occurrence of complications as the dependent variable and pretreatment stoma as the independent variable

Complication analysis	Unadjusted OR (95% c.i.)	Adjusted OR (95% c.i.)†
Complications greater than or equal to Clavien–Dindo grade II between diagnosis and resection	7.12 (2.52,20.15)	6.71 (2.17,20.76)
Sensitivity analysis, non-resected patients excluded	6.53 (2.21,19.32)	6.67 (1.92,23.10)
Sensitivity analysis, only complications greater than or equal to Clavien–Dindo grade IIIa	10.58 (3.33,33.60)	12.26 (3.14,47.92)
Complications greater than or equal to Clavien–Dindo grade II within 30 days following resection	2.70 (0.89,8.17)	2.05 (0.62,6.81)

*One patient intended for long-course radiotherapy ultimately received short-course radiotherapy due to complications following the stoma surgery. †Adjustment for age, sex, American Society of Anesthesiologists fitness grade, and clinical tumour stage.

Complications within 30 days following resection surgery are shown in *Table 4*. There were no statistically significant differences in complication incidence and severity between the groups overall (*P* = 0.059), although the proportion of patients experiencing minor or no complications was higher in the control group. Regression analysis resulted in an adjusted non-significant OR of 2.05 (95% c.i. 0.62 to 6.81) for the stoma group, in comparison with the control group.

**Table 4:** Complications within 30 days following rectal cancer resection in the 96 patients who underwent surgery following long-course* neoadjuvant therapy

Clavien–Dindo grade	Patients without a stoma before neoadjuvant therapy, *n* = 76	Patients with a stoma before neoadjuvant therapy, *n* = 20
<II	36 (47.4)	5 (25.0)
II	24 (31.6)	8 (40.0)
IIIa	7 (9.2)	3 (15.0)
IIIb	8 (10.5)	2 (10.0)
IVa	0 (0.0)	1 (5.0)
IVb	0 (0.0)	0 (0.0)
V	1 (1.3)	1 (5.0)*

Values are *n* (%). Only the highest graded complication for each patient was noted. Mann–Whitney *U* test: *P* = 0.059. *This patient was intended for long-course radiotherapy, but ultimately received short-course radiotherapy due to complications following the stoma surgery.

### Time to treatment and overall survival

**Fig. 2:**
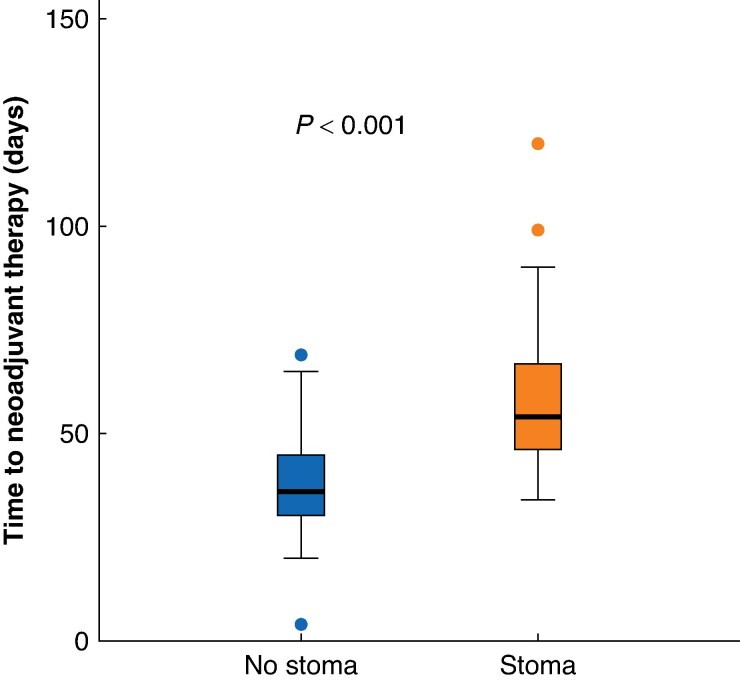
Box plots showing time from diagnosis to neoadjuvant treatment in 105 patients with rectal cancer who received long-course* neoadjuvant therapy, stratified by placement of a pretreatment stoma *One patient in the stoma group was intended for long-course radiotherapy, but ultimately received short-course radiotherapy due to complications following the stoma surgery.

**Fig. 3:**
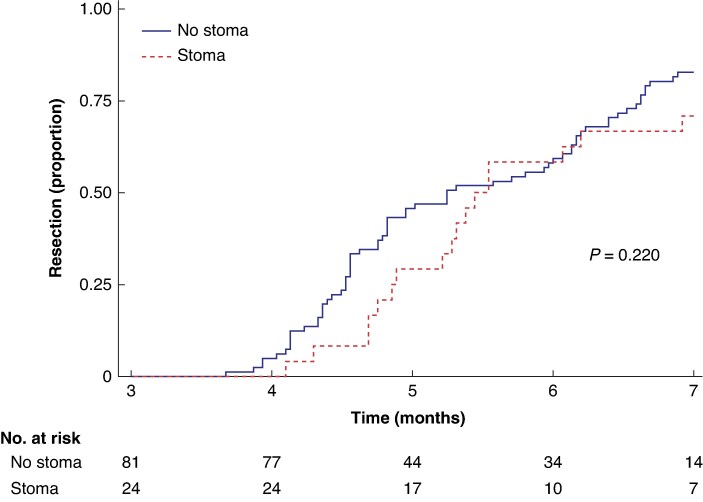
Kaplan–Meier curve showing time from diagnosis to resection surgery in 105 patients with rectal cancer who received long-course* neoadjuvant therapy, stratified by placement of a pretreatment stoma Note that the curve begins at 3 months and is truncated when the number at risk drops below one-third of the starting number. *One patient in the stoma group was intended for long-course radiotherapy, but ultimately received short-course radiotherapy due to complications following the stoma surgery.

As shown in *Fig. 2*, the time to start of neoadjuvant treatment was significantly longer in the stoma group (*t* test: *P* < 0.001). The median time was 54 (i.q.r. 46–67) days in the stoma group compared with 36 (i.q.r. 30–45) days in the control group. Using linear regression, this corresponds to an unadjusted mean difference of 21 (95% c.i. 14 to 27) days. Concerning time to resection surgery (*Fig. 3*), it took a median of 163 (95% c.i. 147 to 208) and 158 (95% c.i. 145 to 182) days for the stoma group and the control group respectively. This difference was not significant (log rank test: *P* = 0.220). Regarding survival, the stoma group performed significantly worse than the control group, as illustrated in *Fig. S1* (log rank test: *P* = 0.015). The median survival after diagnosis was 4.7 (95% c.i. 2.5 to 7.9) years in the stoma group and 12.2 (95% c.i. 5.7 to not estimable) years in the control group.

As shown in *Table 5*, confounder adjustment using linear and Cox regression resulted in relatively similar unadjusted and adjusted results for time to neoadjuvant treatment and time to resection surgery respectively. Regarding overall survival, however, adjustment rendered the estimate non-significant.

**Table 5:** Multivariable linear and Cox regression analyses: unadjusted and adjusted time differences and HRs with 95% confidence intervals for 105 patients with rectal cancer who received long-course* neoadjuvant therapy, using time to event as the dependent variable and pretreatment stoma as the independent variable

Time-to-event analysis	Unadjusted (95% c.i.)	Adjusted (95% c.i.)†
Neoadjuvant treatment (linear regression, mean time difference in days)	21 (14,27)	21 (14,27)
Resection surgery (Cox regression, HR)	0.74 (0.45,1.21)	0.70 (0.42,1.18)
Death (Cox regression, HR)	2.07 (1.14,3.76)	1.71 (0.93,3.14)

*One patient intended for long-course radiotherapy ultimately received short-course radiotherapy due to complications following the stoma surgery. †Adjustment for age, sex, American Society of Anesthesiologists fitness grade, and clinical tumour stage.

### Trend of creating a pretreatment stoma

**Fig. 4:**
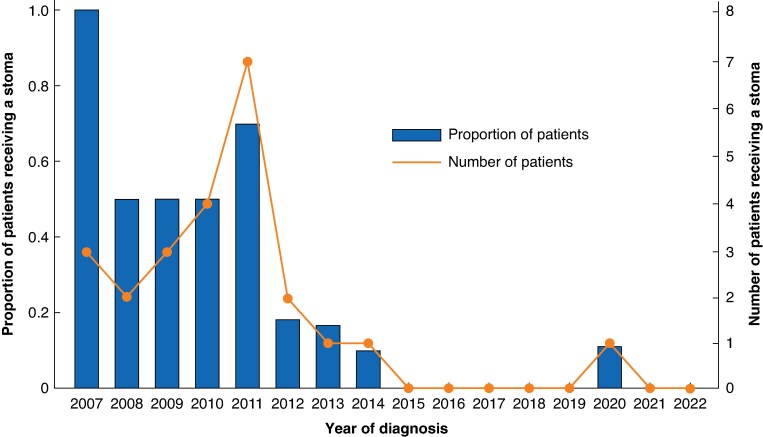
Graph showing the trend of establishing a planned defunctioning stoma before long-course* neoadjuvant therapy for rectal cancer in Region Västerbotten, Sweden, between 2007 and 2022 *One patient who received a pretreatment stoma was intended for long-course radiotherapy, but ultimately received short-course radiotherapy due to complications following the stoma surgery.

As shown in *Fig. 4*, nearly all of the included patients who received a stoma before long-course neoadjuvant therapy were diagnosed in the earlier half of the study interval. With the exception of 2011, which exhibited a notable increase in both the proportion and number of pretreatment stomas utilized, there was a consistent decrease in stoma usage up to 2015. After that, only one pretreatment stoma was created amongst the included patients.

## Discussion

This study indicates that the creation of a defunctioning stoma before long-course neoadjuvant therapy in rectal cancer is associated with a significantly higher incidence of complications, particularly those greater than or equal to Clavien–Dindo grade IIIa. Furthermore, high-grade complications were often directly related to the stoma surgery itself. The major complication rate following resection surgery was similar between groups, indicating that the difference in such complications before resection was unlikely to be due to confounding patient factors, as patients with clear indications for a defunctioning stoma were excluded. This is corroborated by the multivariable regression analysis that was performed. Nonetheless, pretreatment stomas may have benefits as well, which is demonstrated by the three patients in the control group who eventually suffered large bowel obstruction. Clinical judgement is instrumental when determining whom to defunction, due to the scarcity of scientific evidence. Before proper guidelines can be established, further research is necessary.

The increased time to start of neoadjuvant therapy in the stoma group was expected, given the added surgical procedure. However, the mean difference in time to treatment was merely 21 days, which might not be clinically relevant. Furthermore, this time difference seems to equalize as treatment progresses, as the time interval from diagnosis to resection surgery was similar across groups.

Receiving a pretreatment defunctioning stoma was associated with worse survival in the unadjusted analysis, although the exact relationship to the stoma surgery remains uncertain. It seems unlikely that pre-resection complications are responsible for the reduced survival, especially as a pretreatment stoma did not influence the complication rate following resection. A more plausible explanation is confounding, supported by the multivariable regression analysis that attenuated the survival differences and rendered the finding non-significant. This could suggest that the covariates chosen for the analysis, while few, were appropriate for the sample, thereby reinforcing the findings regarding complications discussed above.

The present study demonstrates that the practice of establishing a stoma before neoadjuvant therapy in rectal cancer has largely disappeared in Region Västerbotten, Sweden. The underlying reasons for this shift are not entirely known. However, the change seems to coincide with the introduction of total neoadjuvant therapy in around 2012. The short, but intensive, radiotherapy in the RAPIDO regimen might have caused the multidisciplinary team meetings to assume an accelerated tumour regression compared with conventional chemoradiation, with an accompanying lower perceived risk of bowel obstruction.

There are limitations to the present study. Its observational design makes it vulnerable to selection bias, as patients were not randomly assigned to either group. Also, the retrospective nature of the study prevents full assessment of the treating physician’s decision-making process. Finally, the sample size is limited, with an uneven distribution across groups. Although statistical analyses were employed to minimize confounding, the small sample size only allowed adjustments for the most important covariates. Nonetheless, there are strengths to the study as well. To the best of the authors’ knowledge, it is the largest study to date covering this topic, despite its modest size. Furthermore, it is the only study providing a detailed account of complication rates and severity in these patients, which could be valuable in areas where pretreatment stomas are still in widespread use.

A recent 5-year follow-up of the RAPIDO trial, where short-course radiotherapy followed by four to six cycles of chemotherapy was compared with conventional long-course chemoradiation, suggested that the RAPIDO regimen results in an increased risk of locoregional recurrence^14^. Consequently, long-course chemoradiation might regain popularity in regions where the RAPIDO regimen is predominantly used today, thus further increasing the relevance of the present study.

There are only a handful of reports discussing defunctioning stomas established before neoadjuvant therapy in rectal cancer, all of which are small-scale, observational studies. Anderson *et al*.^7^ published one such study in 2015. In this study, 103 patients were assessed regarding time to treatment, complications, and survival. However, only 13 patients received a pretreatment stoma and those presenting with urgent bowel-related symptoms were included. Moreover, the complication assessment did not include any severity grading. Thus, this study is of limited use for evaluating pretreatment stomas as a prophylactic measure in patients without obstructive symptoms. A smaller study published by Parnaby *et al*.^15^ in 2008 had similar issues with its complication assessment and neither study was population-based. The studies suggest that pretreatment stomas are mandated for patients presenting with urgent bowel-related symptoms or when the tumour cannot be traversed during endoscopy, respectively. However, the latter recommendation can be questioned, as two more recent studies have shown that pretreatment stomas may not be necessary, even in endoscopically obstructing rectal cancers^8,16^.

The pretreatment stoma strategy seems to originate from the belief that irradiation causes tumour oedema, which could potentially lead to bowel obstruction. Additionally, such stomas may help alleviate some of the side effects of chemotherapy, namely severe diarrhoea that might hinder further treatment. However, both the present report and previous studies indicate that these concerns might not be as prevalent as anticipated. On the contrary, the surgical procedure of creating a stoma appears to carry greater risks than some surgeons or oncologists realize.

In conclusion, although some patients with rectal cancer may benefit from a defunctioning stoma before long-course neoadjuvant therapy, the majority are exposed to a higher risk of complications, potentially without gaining tangible advantages. The challenge lies in identifying which patients fall into each category. Interestingly, the use of prophylactic stomas has declined in Region Västerbotten, Sweden, perhaps due to the introduction of the RAPIDO regimen. Until further studies are conducted, this change is likely for the better.

## Supplementary Material

zrae169_Supplementary_Data

## Data Availability

Upon reasonable request, data and methodology can be shared. This also applies to the registry-based data used in the present report, although access to such data might be subject to external review by the Swedish Colorectal Cancer Registry steering committee.
