# Supplementary material for: Stoma versus no stoma prior to long-course neoadjuvant therapy in rectal cancer
Source: BJS Open. 2025 Mar 18;9(2):zrae169. doi: 10.1093/bjsopen/zrae169 (PMC11913605; doi:10.1093/bjsopen/zrae169)
Supplement: zrae169_Supplementary_Data [file zrae169_supplementary_data.docx]

# Gut-wrenching decisions: Stoma or no stoma prior to long-course neoadjuvant therapy in rectal cancer?

#### Authors

Gustav Sandén, M.D. (1), Petrus Vinnars, M.D., Ph.D. (1), Ingrid Ljuslinder, M.D., Ph.D. (2), Johan Svensson, Ph.D. (3), Martin Rutegård, M.D., Ph.D. (1).

#### Affiliations

1. Department of Diagnostics and Intervention, Surgery, Umeå University, Umeå, Sweden.
2. Department of Diagnostics and Intervention, Oncology, Umeå University, Umeå, Sweden.
3. Umeå School of Business, Economics and Statistics, Umeå University, Umeå, Sweden.

#### Correspondence

#### Gustav Sandén, M.D. (gustav.sanden@umu.se)

#### Department of Diagnostics and Intervention, Surgery, Umeå University

#### SE-901 85 Umeå, Sweden

ORCID: 0000-0002-2519-8020

**Supplementary Materials - Index**

| **Supplementary Figures and Tables** |  |
| --- | --- |
| Figure S1 – Overall survival | *pag. 3* |
|  |  |

**Supplementary Figures and Tables**

**Fig. S1.** Kaplan-Meier curve showing overall survival in 105 rectal cancer patients receiving long-course* neoadjuvant therapy, stratified by placement of a pre-treatment stoma. Note that the graph is limited to visualise only five years.


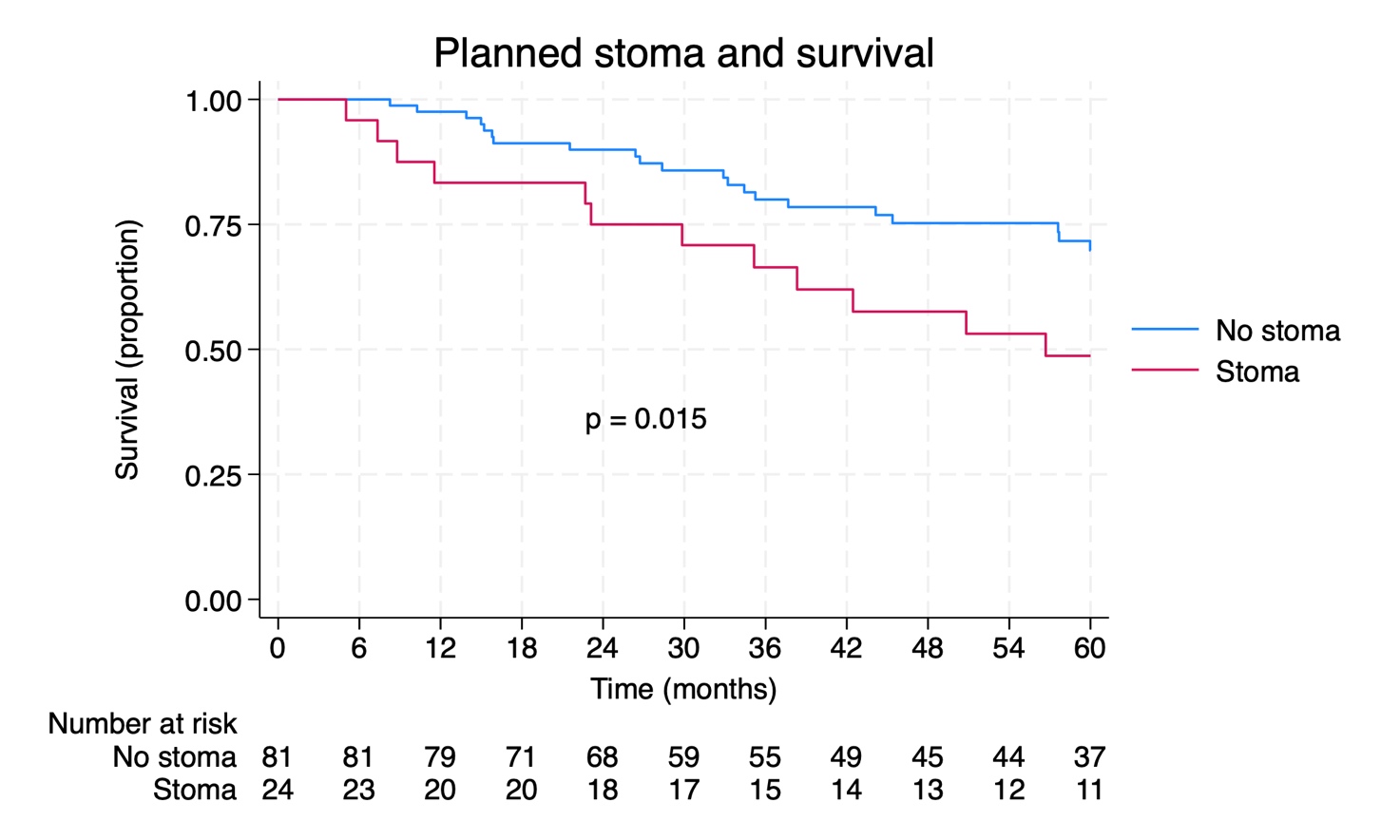


*One patient in the stoma group was intended for long-course radiotherapy, but ultimately received short-course radiotherapy due to complications after the stoma surgery.
